# The erbB3- and IGF-1 receptor-initiated signaling pathways exhibit distinct effects on lapatinib sensitivity against trastuzumab-resistant breast cancer cells

**DOI:** 10.18632/oncotarget.6404

**Published:** 2015-11-26

**Authors:** Hui Lyu, Xiao He Yang, Susan M. Edgerton, Ann D. Thor, Xiaoying Wu, Zhimin He, Bolin Liu

**Affiliations:** ^1^ Cancer Research Institute and Affiliated Cancer Hospital, Guangzhou Medical University, Guangzhou, Guangdong, China; ^2^ Department of Pathology, School of Medicine, University of Colorado Anschutz Medical Campus, Aurora, CO, USA; ^3^ Julius L. Chambers Biomedical/Biotechnology Research Institute, North Carolina Central University, Kannapolis, NC, USA; ^4^ Department of Pathology, Xiangya Hospital, School of Basic Medical Science, Central South University, Changsha, China

**Keywords:** ErbB3, IGF-1R, lapatinib, trastuzumab, resistance

## Abstract

Both erbB3 and IGF-1 receptor (IGF-1R) have been shown to play an important role in trastuzumab resistance. However, it remains unclear whether erbB3- and IGF-1R-initiated signaling pathways possess distinct effects on the sensitivity of lapatinib, a dual tyrosine kinase inhibitor against both EGFR and erbB2, in trastuzumab-resistant breast cancer. Here, we show that the trastuzumab-resistant SKBR3-pool2 and BT474-HR20 breast cancer sublines, as compared the parental SKBR3 and BT474 cells, respectively, exhibit refractoriness to lapatinib. Knockdown of erbB3 inhibited Akt in SKBR3-pool2 and BT474-HR20 cells, significantly increased lapatinib efficacy, and dramatically re-sensitized the cells to lapatinib-induced apoptosis. In contrast, specific knockdown of IGF-1R did not alter the cells' responsiveness to lapatinib. While the levels of phosphorylated Src (P-Src) were reduced upon IGF-1R downregulation, the P-Akt levels remained unchanged. Furthermore, a specific inhibitor of Akt, but not Src, significantly enhanced lapatinib-mediated anti-proliferative/anti-survival effects on SKBR3-pool2 and BT474-HR20 cells. These data indicate that erbB3 signaling is critical for both trastuzumab and lapatinib resistances mainly through the PI-3K/Akt pathway, whereas IGF-1R-initiated Src activation results in trastuzumab resistance without affecting lapatinib sensitivity. Our findings may facilitate the development of precision therapeutic regimens for erbB2-positive breast cancer patients who become resistant to erbB2-targeted therapy.

## INTRODUCTION

One of the most well characterized oncogenes involved in breast carcinogenesis is *erbB2* (or *HER2*/*neu*). Gene amplification/overexpression of e*rbB2* is observed in approximately 25–30% of invasive breast cancers and significantly associated with a worse prognosis [1, 2]. The erbB2 receptor has no known ligand. It may become activated by overexpression via either homodimerization or heterodimerization with another receptor tyrosine kinase (RTK). ErbB2 is therefore an ideal target for breast cancer treatment. Lapatinib (or Tykerb) is a small molecule inhibitor, and dual targets both the epidermal growth factor receptor (EGFR) and erbB2. Because the majority of erbB2-overexpressing (erbB2-positive) breast cancer cells express little or basal levels of EGFR, lapatinib mainly inhibits erbB2 kinase activity (intracellular domain) in erbB2-positive breast cancers. Another erbB2-targeted therapy, trastuzumab (Herceptin) is a humanized monoclonal antibody (Ab) binding to the extracellular domain of erbB2. Both trastuzumab and lapatinib have been successfully used in clinic to treat early and metastatic breast cancer (MBC) patients with erbB2-positive tumors [3–8]. However, both *de novo* and acquired resistance to these agents frequently occurs, representing a significant clinical problem [9–12].

A number of studies suggest that lapatinib resistance arises via mechanisms similar to those contributing to trastuzumab resistance. For instance, activation of the signaling pathways initiated by other erbB receptors, such as EGFR and erbB3, can impair the anti-proliferative effects of lapatinib [13–16]. Compensatory signaling activation resulting from other RTKs outside of the erbB family, such as AXL, may also cause resistance to lapatinib [17]. In addition, upregulation of survivin, the smallest member of the inhibitor of apoptosis (IAP) family, has been identified as a contributor to lapatinib resistance [18]. Some non-overlapping mechanisms of resistance to trastuzumab and lapatinib likely exist in erbB2-positive breast cancers, as lapatinib has been approved by the FDA to treat erbB2-positive MBC that has progressed on trastuzumab-based therapy [19]. In fact, increasing evidence suggests that lapatinib and trastuzumab do not share common mechanisms of resistance, since lapatinib has activity in trastuzumab-resistant breast cancer [20–23]. These conclusions are supported by clinical data showing improved outcomes derived from inflammatory breast cancer patients [24]. For example, the PI-3K/Akt signaling pathway is a major determinant of trastuzumab resistance in breast cancers [25], whereas its role in lapatinib resistance remains controversial. One study has shown that loss of PTEN and the resulting activation of PI-3K/Akt signaling lead to lapatinib resistance, and this can be reversed by the mTOR/PI-3K inhibitor NVP-BEZ235 [26]. Others report that activation of PI-3K/Akt signaling confers resistance to trastuzumab but not lapatinib [27, 28] and lapatinib exerts anti-tumor activity in a PTEN independent manner [29]. Wang *et al* have shown that estrogen receptor (ER) and erbB2 reactivation play important roles in the differential resistance of trastuzumab as compared to lapatinib [30].

A recent report has identified the non-receptor tyrosine kinase Src as a crucial mediator of trastuzumab resistance in erbB2-positive breast cancers [31]. It shows that loss of PTEN or overexpression of another RTK, such as the insulin-like growth factor-I receptor (IGF-1R), EGFR, or erbB3 induces activation of Src and thereby promotes trastuzumab resistance in a PI-3K/Akt-dependent or -independent manner [32]. These observations have been supported by the studies indicating that administration of erythropoietin induces Jak2-mediated activation of Src and PTEN inactivation, reducing trastuzumab efficacy [33]. Thus, Src activation appears to be a key mechanism of trastuzumab resistance and predicts for poor prognosis mainly in erbB2-positive/ER-negative breast cancer [34]. Several studies have also found that activation of Src causes lapatinib resistance [35, 36], more specifically activated Src is upregulated in β1-integrin- and mTORC1-mediated resistance to lapatinib in erbB2-positive breast cancer cells [37, 38]. However, whether Src activation may cause cross-resistance to both trastuzumab and lapatinib remains unclear. It is not known whether the activation of Src in trastuzumab-resistant breast cancer observed by Zhang *et al* [31] and Liang *et al* [33] affects lapatinib sensitivity.

Finally, both erbB3- and IGF-1R-initiated signaling pathways have been shown to be involved in trastuzumab resistance [39–41]. We previously reported that the erbB2 receptor simultaneously interacted with erbB3 and IGF-1R to form a heterotrimeric complex in trastuzumab-resistant breast cancer cells [42]. This interaction enhanced activation of the PI-3K/Akt signaling and Src kinase. Specific knockdown of either erbB3 or IGF-1R significantly reversed this resistance capacity, suggesting that both erbB3 and IGF-1R contributed to trastuzumab resistance [42]. Here, we take advantage of the same cell models to determine if the trastuzumab-resistant breast cancer cells become refractory to lapatinib, and to explore whether erbB3- and IGF-1R-initiated signaling pathways differentially modulate lapatinib sensitivity.

## RESULTS

### Trastuzumab-resistant breast cancer sublines are less sensitive than the parental lines to lapatinib-induced growth inhibition and apoptosis

**Figure 1:**
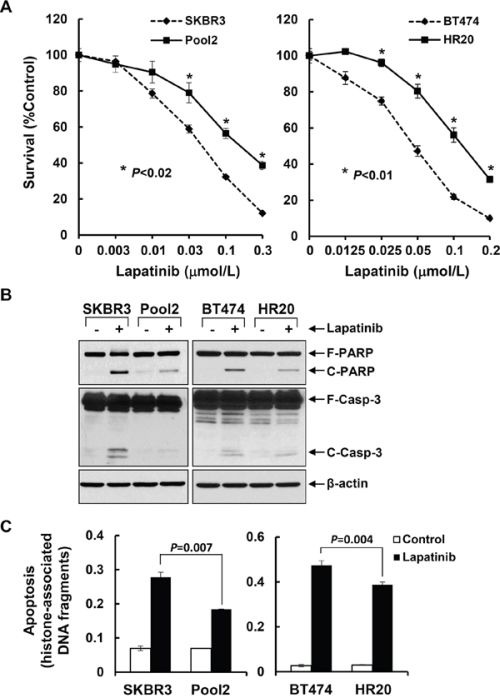
Trastzumab-resistant breast cancer cell lines are significantly insensitive than their parental lines to lapatinib-mediated growth inhibition and apoptosis **A.** SKBR3 and Pool2 or BT474 and HR20 cells were plated onto 96-well plates and incubated at 37°C with 5% CO2. After 24 hr, the culture medium was replaced with 0.1 ml fresh medium containing 0.5% FBS or the same medium containing the indicated concentrations of lapatinib for another 72 hr. The percentages of surviving cells from each cell line relative to controls, defined as 100% survival, were determined by reduction of MTS. *Bars*, SD. Data show a representative of three independent experiments. **B & C.** The same cells were untreated or treated with lapatinib (0.1 μmol/L) for 24 hr. Cells were collected and subjected to western blot analyses of PARP (F-PARP, full length PARP; C-PARP, cleaved PARP), caspase-3 (F-Casp-3, full length caspase-3; C-Casp-3, cleaved caspase-3), or β-actin (B); or a specific apoptosis ELISA (C). *Bars*, SD.

To determine the lapatinib efficacy against trastuzumab-resistant breast cancer cells, we used SKBR3-pool2 and BT474-HR20 sublines derived from SKBR3 and BT474 breast cancer cells, respectively. As compared to the parental lines, the trastuzumab-resistant SKBR3-pool2 and BT474-HR20 cells were significantly less sensitive to lapatinib-induced growth inhibition (Figure 1A). The IC_50_ values of lapatinib were approximately 0.043 μmol/L ± 0.003 in SKBR3 cells *vs* 0.15 μmol/L ± 0.003 in SKBR3-pool2 cells, and 0.054 μmol/L ± 0.009 in BT474 cells *vs* 0.12 μmol/L ± 0.006 in BT474-HR20 cells (*P* < 0.05). While lapatinib induced profound PARP cleavage and activation of caspase-3, the hallmarks of apoptosis, and histone-associated DNA fragmentation in both SKBR3 and BT474 cells, less PARP cleavage and caspase-3 activation and significantly reduced DNA fragments were observed in their trastuzumab-resistant counterparts upon lapatinib treatment (Figure 1B & 1C). These data indicate that trastuzumab-resistant breast cancer cells are refractory to lapatinib-induced growth inhibition and apoptosis.

### Specific knockdown of erbB3, but not IGF-1R, inhibits proliferation of the trastuzumab-resistant breast cancer cells associated with cell cycle G1 arrest and significantly promotes lapatinib-mediated growth inhibition and apoptosis

**Figure 2:**
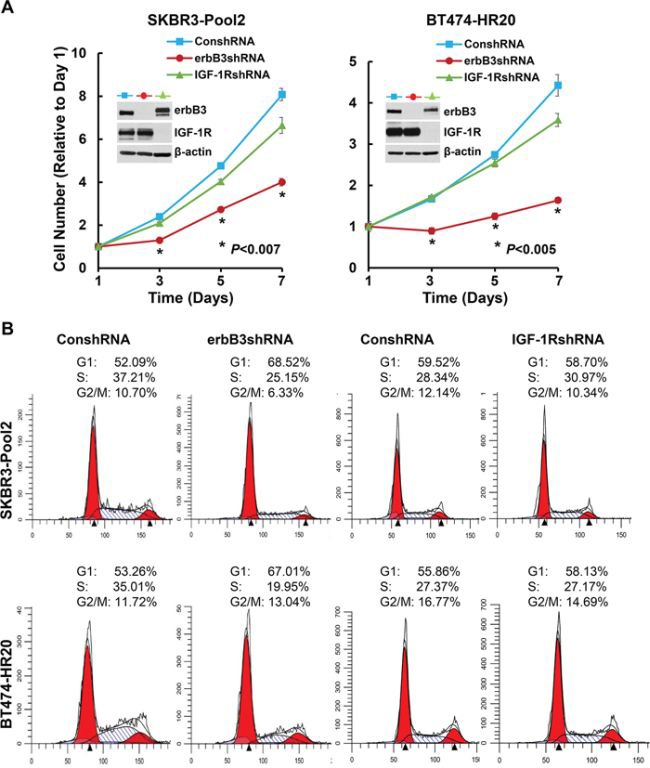
Specific knockdown of erbB3, but not IGF-1R, suppresses proliferation of trastzumab-resistant breast cancer cells associated with cell cycle G1 arrest SKBR3-Pool2 and BT474-HR20 cells infected with lentivirus containing either ConshRNA or erbB3/IGF-1R shRNA (erbB3shRNA or IGF-1RshRNA) were subjected to the following experiments. **A.** cell proliferation analysis by MTS assays. 2 × 10^3^ cells were plated onto 96-well plate. The cell number changes relative to Day 1 were determined by reduction of MTS. Values represent the mean ± standard deviation (*n* = 5) from a representative experiment performed three times with similar results. The inserts show western blot assays indicating the specific downregulation of erbB3 or IGF-1R in both cell lines. **B.** cell cycle analysis by flow cytometry. Cells were harvested and fixed with 70% ethanol overnight. Cells were then stained for total DNA content with a solution containing 50 μg/ml propidium iodide and 100 μg/ml RNase I in PBS for 30 min at 37°C. Cell cycle distribution was analyzed by a flow cytometer.

**Figure 3:**
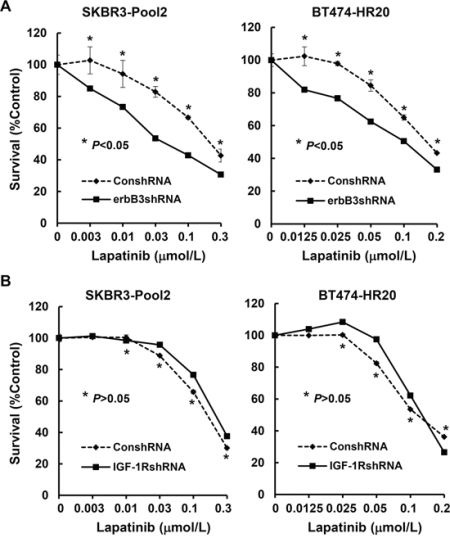
Specific knockdown of erbB3, but not IGF-1R, re-sensitizes the trastzumab-resistant breast cancer cells to lapatinib-mediated growth inhibition SKBR3-Pool2 and BT474-HR20 cells infected with lentivirus containing either ConshRNA or erbB3shRNA/1RshRNA were plated onto 96-well plates. After 24 hr, the culture medium was replaced with 0.1 ml fresh medium containing 0.5% FBS or the same medium containing the indicated concentrations of lapatinib for another 72 hr. The percentages of surviving cells from each cell line relative to controls, defined as 100% survival, were determined by reduction of MTS. *Bars*, SD. Data show a representative of three independent experiments.

We have reported that both erbB3- and IGF-1R-initiated signaling pathways contribute to trastuzumab resistance [42]. Thus, we examined whether the two receptors might also modulate the inhibitory effects of lapatinib on SKBR3-pool2 and BT474-HR20 cells. The cells were infected with the lentivirus containing control, *erbB3*, or *IGF-1R* shRNA for specific gene silencing. We first performed western blot assays, showing that the shRNA sequences we used were specific to knockdown their corresponding receptors (Figure 2A, inserts). While knockdown of erbB3 significantly reduced cell growth, specific downregulation of IGF-1R had no effect on proliferation of the cells (Figure 2A). Flow cytometry analysis revealed that the *erbB3* shRNA (erbB3shRNA) increased cell population in G1 phase of the cell cycle and decreased the percentage of cells in S phase, whereas the shRNA for *IGF-1R* (IGF-1RshRNA) did not alter cell cycle distribution as compared to control shRNA (Figure 2B). Furthermore, specific knockdown of erbB3 expression was able to significantly re-sensitize the resistant cells to lapatinib-mediated growth inhibition (Figure 3A). This dramatic reduction might also be attributed to the significant inhibitory effects-caused by specific knockdown of erbB3 expression (Figure 2A). However, specific knockdown of IGF-1R expression showed no significant impact on lapatinib-induced inhibitory effects on SKBR3-pool2 and BT474-HR20 cells (Figure 3B).

**Figure 4:**
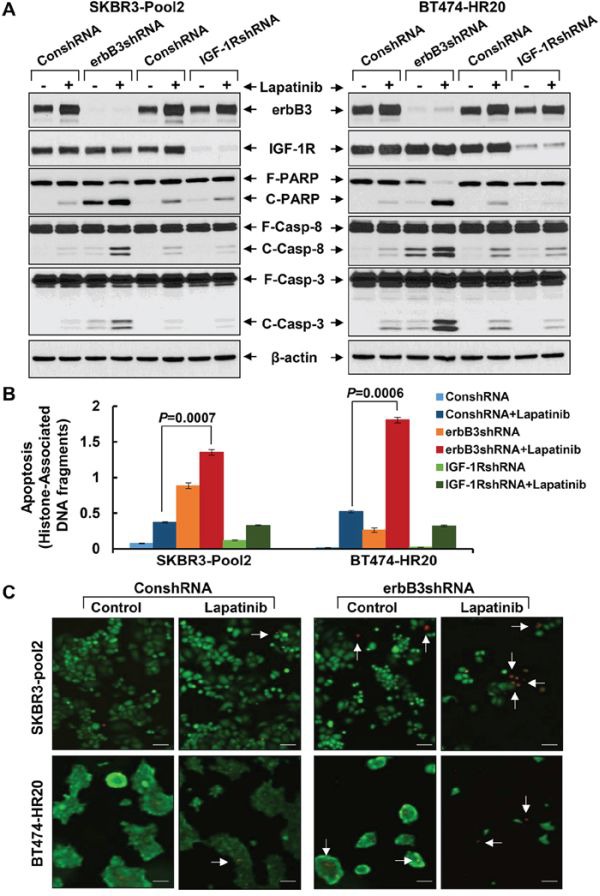
Specific knockdown of erbB3, but not IGF-1R, markedly enhances lapatinib-induced apoptosis in trastzumab-resistant breast cancer cells SKBR3-Pool2 and BT474-HR20 cells infected with lentivirus containing either ConshRNA or erbB3/IGF-1R shRNA (erbB3shRNA or IGF-1RshRNA). The cells were then untreated or treated with lapatinib (0.1 μmol/L) for 24 hr and subjected to the following experiments. **A & B.** Western blot analyses of erbB3, IGF-1R, PARP (F-PARP, full length PARP; C-PARP, cleaved PARP), caspase-8 (F-Casp-8, full length caspase-8; C-Casp-8, cleaved caspase-8), caspase-3 (F-Casp-3, full length caspase-3; C-Casp-3, cleaved caspase-3), or β-actin (A); or a specific apoptosis ELISA (B). *Bars*, SD. **C.** Live/dead cell staining. Cells freshly stained with Live/Dead Imaging kit. Red indicated the dead cells (white arrow).

We next investigated the influence of erbB3 receptor and IGF-1R on lapatinib-induced apoptosis in trastuzumab-resistant breast cancer cells. The efficiency and specificity of the *erbB3* shRNA and *IGF-1R* shRNA were demonstrated previously [42] and further confirmed in the current study (Figures 2A & 4A). While lapatinib treatment did not affect the expression levels of IGF-1R, it clearly increased erbB3 levels in both SKBR3-pool2 and BT474-HR20 cells (Figure 4A). This observation is in agreement with a recent report showing that lapatinib can induce a compensatory upregulation of erbB3 in erbB2-positive breast cancer cells [14]. More importantly, specific knockdown of erbB3 significantly enhanced lapatinib-induced apoptosis, evidenced by increased PARP cleavage, activation of caspase-8 and -3, and histone-associated DNA fragmentation (Figure 4A & 4B). In contrast, IGF-1R knockdown had no effect on lapatinib-induced apoptosis. Additional studies with live/dead imaging assays revealed that the *erbB3* shRNA in combination with lapatinib exhibited a profound cell killing activity as compared to either lapatinib treatment or *erbB3* shRNA alone (Figure 4C). Collectively, our studies demonstrate that erbB3 receptor and IGF-1R differentially modulate lapatinib sensitivity in trastuzumab-resistant breast cancer cells; and specific knockdown of erbB3, but not IGF-1R, significantly promotes lapatinib-mediated growth inhibition and apoptosis.

### Downregulation of erbB3 mainly reduces the levels of phosphorylated Akt (P-Akt), whereas IGF-1R knockdown leads to a decrease of P-Src levels

**Figure 5:**
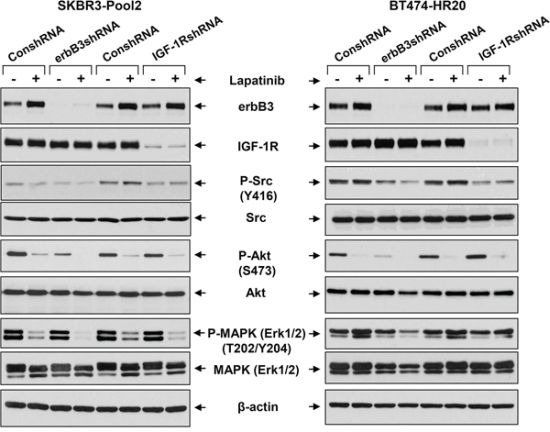
The erbB3 receptor and IGF-1R initiates activation of distinct downstream signaling pathways in trastzumab-resistant breast cancer cells SKBR3-Pool2 and BT474-HR20 cells infected with lentivirus containing either ConshRNA or erbB3/IGF-1R shRNA (erbB3shRNA or IGF-1RshRNA). The cells were then untreated or treated with lapatinib (0.1 μmol/L) for 24 hr. Cells were collected and subjected to western blot analyses of erbB3, IGF-1R, P-Src (Y416), Src, P-Akt (S473), Akt, P-MAPK (Erk1/2) (T202/Y204), MAPK (Erk1/2), or β-actin.

Enhanced activation of the downstream signaling pathways, including PI-3K/Akt and Src kinase, were observed upon the hetrotrimerization of erbB2/erbB3/IGF-1R in SKBR3-pool2 and BT474-HR20 cells. Specific inhibitors of either Akt or Src were able to abrogate the trastuzumab resistant phenotype [42], consistent with recent studies showing that both PI-3K/Akt signaling and Src kinase are critically involved in trastuzumab resistance [25, 31, 32]. We wondered whether erbB3 receptor and IGF-1R equally initiated activation of the PI-3K/Akt signaling and Src kinase, which might also influence the lapatinib sensitivity of trastuzumab-resistant cells. We found that specific knockdown of erbB3 dramatically decreased the levels of P-Akt, and to a less extent P-Src, in both SKBR3-pool2 and BT474-HR20 cells. In contrast, IGF-1R knockdown only gave rise to a reduction of P-Src (Figure 5). Treatment with lapatinib alone dramatically reduced P-Akt levels in both cell lines, inhibited P-MAPK (Erk1/2) in SKBR3-pool2 cells; and it had a minor effect on P-Src. Interestingly, lapatinib combined with downregulation of erbB3, but not IGF-1R, exhibited a most profound inhibition on P-Akt, P-Src, and P-MAPK (Erk1/2) in both cell lines. Our data suggest that erbB3 and IGF-1R initiate distinct signaling pathways contributing to trastuzumab resistance - erbB3 activates both PI-3K/Akt signaling and Src kinase, whereas IGF-1R mainly elicits Src activation.

### Inhibition of Akt, but not Src, significantly enhances lapatinib-induced growth inhibition, long-term suppressive effects on colony formation, and apoptosis in trastuzumab-resistant breast cancer cells

**Figure 6:**
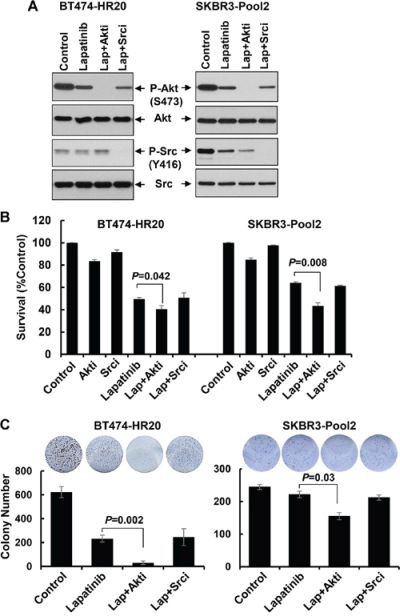
Specific inhibition of Akt, but not Src, significantly enhances lapatinib-mediated growth inhibition and long-term suppressive effects on colony formation **A.** SKBR3-Pool2 and BT474-HR20 cells were untreated or treated with lapatinib (0.1 μmol/L) or lapatinib combined with Akt/Src inhibitor (Lap+Akti/Lap+Srci) for 24 hr. Cells were collected and subjected to western blot analyses of P-Akt (S473), Akt, P-Src (Y416), Src. **B.** SKBR3-Pool2 and BT474-HR20 cells were plated onto 96-well plates. After 24 hr, the culture medium was replaced with 0.1 ml fresh medium containing 0.5% FBS or the same medium containing Akti (0.5 μmol/L), Srci (0.2 μmol/L), lapatinib (0.1 μmol/L), lapatinib+Akti, lapatinib+Srci for another 72 hr. The percentages of surviving cells from each cell line relative to controls, defined as 100% survival, were determined by reduction of MTS. *Bars*, SD. Data show a representative of three independent experiments. **C.** colony formation assays. 1 × 10^3^ SKBR3-Pool2 or BT474-HR20 cells were seeded onto 12-well plates. Cells were cultured with 0.1 ml fresh medium containing 0.5% FBS or the same medium containing lapatinib (0.1 μmol/L), lapatinib+Akti (0.5 μmol/L), lapatinib+Srci (0.2 μmol/L) for 2 weeks. The medium changed very 3 day. *Bars*, SD. Data show a representative of three independent experiments.

**Figure 7:**
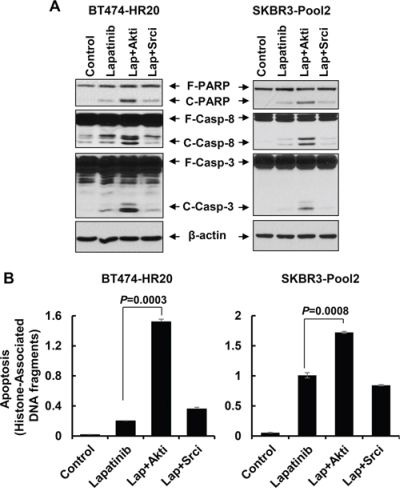
The Akt inhibitor, but not Src inhibitor, dramatically potentiates lapatinib-induced apoptosis in trastzumab-resistant breast cancer cells SKBR3-Pool2 and BT474-HR20 cells untreated or treated with lapatinib (0.1 μmol/L) or lapatinib combined with Akt/Src inhibitor (Lap+Akti/Lap+Srci) for 24 hr were subjected to the following experiments. **A & B.** Western blot analyses of PARP (F-PARP, full length PARP; C-PARP, cleaved PARP), caspase-8 (F-Casp-8, full length caspase-8; C-Casp-8, cleaved caspase-8), caspase-3 (F-Casp-3, full length caspase-3; C-Casp-3, cleaved caspase-3), or β-actin (A); or a specific apoptosis ELISA (B). *Bars*, SD.

We next focused on studying if the PI-3K/Akt signaling and Src kinase also influenced the refractoriness of lapatinib displayed by the trastuzumab-resistant cells. Specific inhibitor of either Akt or Src was used. Our previous studies [43] showed that the Akt1/2 kinase inhibitor (Akti) at 0.5 – 1.0 μmol/L was sufficient to reduce the P-Akt levels in SKBR3 cells with ectopic expression of erbB3. Since we had no experience with the Src inhibitor (Srci) Saracatinib, we first performed preliminary studies and discovered that the Srci at 0.2 μmol/L clearly decreased P-Src, whereas it had no effect on P-Akt (Supplementary Figure S1). Thus, 0.5 μmol/L of Akti and 0.2 μmol/L of Srci were used in the following studies. Treatment with lapatinib alone decreased P-Akt levels in both BT474-HR20 and SKBR3-pool2 cells, and reduced P-Src only in SKBR3-pool2 cells (Figure 6A). Lapatinib in combination with Akti eliminated P-Akt and had little effect on P-Src in both cell lines, whereas lapatinib in combination with Srci abolished P-Src and resulted in no further reduction of P-Akt than lapatinib alone (Figure 6A). These data suggest that the inhibitors we used were potent and specific. Interestingly, while Akti or Srci alone had little effect on cell growth (Figure 6B), Akti significantly enhanced lapatinib-mediated growth inhibition and long-term suppression of colony formation in both BT474-HR20 and SKBR3-pool2 cells. In contrast, the combinations of lapatinib and Srci elicited a similar inhibitory effect as lapatinib alone on cell growth and colony formation (Figure 6B & 6C). Further studies showed a concentration dependent additional inhibitory effect, i.e. lapatinib plus various concentrations of Akti dramatically shifted the cells' responsive curves as compared to lapatinib alone (Supplementary Figure S2). Moreover, it was the Akti, but not the Srci, that significantly enhanced lapatinib-induced PARP cleavage, activation of caspase-8 and -3 (Figure 7A), and histone-associated DNA fragments in both cell lines (Figure 7B). Collectively, our data demonstrate that activation of the PI-3K/Akt signaling, not Src kinase, is associated with the reduced sensitivity to lapatinib in trastuzumab-resistant breast cancer cells.

## DISCUSSION

Despite many important findings that have been reported on the underlying mechanisms of resistance to trastuzumab [11] and lapatinib [10], a number of dilemmas remain [44, 45]: 1) It is unclear if resistance mechanisms for all erbB2-targeted therapies, such as trastuzumab and lapatinib, are similar; 2) There are no accurate methods to identify which breast cancer patients may benefit from, or be resistant to erbB2-targeted therapeutics selected; 3) We lack reliable biomarkers to predict the efficacy of trastuzumab and lapatinib against erbB2-positive breast cancer. Thus, more detailed studies on the underlying mechanisms of trastuzumab and lapatinib resistances should not only further our understanding of breast cancer biology, but also provide a basis for rational design of precision medicines to overcome resistance. As a dual tyrosine kinase inhibitor against both EGFR and erbB2 [46], lapatinib has been approved to treat the erbB2-positive breast cancer patients that have progressed on trastuzumab-based regimens [19]. Lapatinib is able to inhibit the PI-3K/Akt and MEK/MAPK signaling [47] and downregulate the expression of survivin [48]. Unfortunately, the efficacy of lapatinib is also compromised by resistance [10, 49]. The molecular mechanisms of lapatinib resistance are not well understood. We have shown that activation of erbB3 and IGF-1R, the downstream PI-3K/Akt signaling, and Src kinase contributes to trastuzumab resistance [42]. Here, we utilized the same cell models and found that the erbB3/PI-3K/Akt signaling pathway, but not IGF-1R/Src activation, influenced the efficacy of lapatinib. We noticed that the concentrations of lapatinib we used were well below the peak plasma concentration (∼1.5 μmol/L) or steady-state concentration (∼0.5 μmol/L) of lapatinib detected in patients [50]. We showed that, by comparison, the trastuzumab-resistant sublines SKBR3-pool2 and BT474-HR20 were significantly less sensitive to lapatinib than their parental SKBR3 and BT474 cells, respectively. Our data simply suggest that trastuzumab-resistant breast cancer cells may exhibit refractory to lapatinib at certain extend. In addition, our studies were carried out under a 2-D cell culture system, in which the cancer cells were directly exposed to lapatinib. In the treatment of patients, it is difficult to tell how much of the plasma lapatinib actually gets into the microenvironment of solid tumors.

Our findings are consistent with the recent discovery that compensatory upregulation of erbB3 may be phosphorylated (activated) by residual erbB2, maintaining signaling through P-Akt. This limits the antitumor activity of lapatinib [14], and the PI-3K hyperactivation results in lapatinib resistance which can be reversed by the mTOR/PI-3K inhibitor NVP-BEZ235 [26]. Our data do not support an earlier report showing that the cytotoxic effects of lapatinib were further enhanced by the IGF-1R blocking Ab alphaIR3 [51]. To further confirm our data, we also tested an anti-IGF-1R Ab - IMC-A12, a fully human monoclonal IgG1 Ab against human IGF-1R, which is currently under clinical trials (https://www.clinicaltrials.gov/ct2/results?term=IMC-A12&Search=Search) to determine whether we could obtain the same results with the IGF-1R knockdown assays (Figure 3). We discovered that IMC-A12 (A12) as well as the Srci were able to significantly enhance trastuzumab-mediated growth inhibition in both BT474-HR20 and SKBR3-pool2 cells, consistent with our previous report [42]. However, neither A12 nor the Srci altered the two cell lines' responsiveness to lapatinib (Supplementary Figure S3). These results support our current data (Figures 3 & 6). Our findings, distinct from the earlier study [51], might be explained by the different Abs used. Another possibility may be due to the developmental process of the trastuzumab-resistant breast cancer cells. SKBR3-pool2 cells were developed by Dr. Esteva at MD Anderson Cancer Center. His laboratory first discovered that IGF-1R formed heterodimerization with erbB2 and later found that the IGF-1R blocking Ab alphaIR3 increased lapatinib-mediated growth inhibition in the resistant cell line [41, 51]. At that time, SKBR3-pool2 cells were maintained at 4 μg/ml of trastuzumab in the cell culture condition, which may represent as the “early stage” of trastuzumab resistance. Since we obtained the cell line from Dr. Esteva, we continuously cultured SKBR3-pool2 cells by increasing the concentrations of trastuzumab (in order to retain the same condition with BT474-HR20 cells). The SKBR3-pool2 cells are now grown at 20 μg/ml of trastuzumab in our cell culture system. It is possible that the phenotype of this “late stage” of trastuzumab resistance may be distinct from that of “early stage” of trastuzumab resistance, because we not only found that activation of IGF-1R signaling and Src kinase did not alter the efficacy of lapatinib against BT474-HR20 and SKBR3-pool2 cells (Figure 3 & Supplementary Figure S3), we also reported that the erbB2 receptor actually interacted with both erbB3 and IGF-1R to form a heterotrimeric complex in the resistant cells [42]. Nonetheless, more detailed studies are needed to carefully examine the characteristics of “early stage” and “late stage” resistance. In addition, our data are also different from a recent study showing that Src is involved in acquired resistance to lapatinib, and Src inhibitor (saracatinib) restores the sensitivity of the resistant cells to lapatinib [35]. The resistant cell line (SKBR3-Lap-R) used in this study was established through long term selection of SKBR3 cells in the presence of lapatinib (gradually increasing the concentrations); and upregulation of CXCR4 had also been shown to play a role in lapatinib resistance of SKBR3-Lap-R cells. No change of CXCR4 expression was found in our trastuzumab-resistant breast cancer cells (data not shown). Additional studies with the Src inhibitor (saracatinib) revealed that inactivation of Src enhanced trastuzumab-, but not lapatinib-mediated growth inhibition in both BT474-HR20 and SKBR3-pool2 cells (Supplementary Figure S3), further confirming our findings (Figure 6 & see ref 42). Thus, it is conceivable to hypothesize that the underlying mechanisms of cross-resistance to lapatinib caused by trastuzumab-resistant breast cancer cells may be different from those of acquired resistance to lapatinib directly.

It is clear that the erbB3/PI-3K/Akt signaling pathway plays a pivotal role in the development of resistance to trastuzumab and lapatinib. The current clinical inhibitors of PI-3K/Akt cannot completely block the signaling pathway [14], and PI-3K inhibition may induce a feedback upregulation of erbB3 [52]. Thus, effective inhibition of erbB3 is thought to be required for optimal antitumor activity of erbB2-targeted therapy. Our recent studies identified the class I HDAC inhibitor entinostat (SNDX-275 or MS-275) as a special agent to selectively inhibit erbB3 leading to a dramatic reduction of P-Akt in erbB2-positive breast cancer cells [53]. It is interesting and in clinical relevance to study if entinostat and lapatinib may exert synergistic or additive anti-proliferative/anti-survival effects on trastuzumab-resistant breast cancer cells.

In summary, trastuzumab-resistant breast cancer cells as compared to their parental controls are refractory to lapatinib. While inhibition of erbB3 or Akt significantly re-sensitizes the cells to lapatinib treatment, inhibition of IGF-1R or Src kinase does not alter the cells' sensitivity to lapatinib. To the best of our knowledge, this is the first report experimentally demonstrating that erbB3- and IGF-1R-initiated signaling pathways differentially modulate lapatinib efficacy against trastuzumab-resistant cells. Our data provide a basis for rational design of novel effective combinatorial regimens to overcome resistance and thereby improve the survival of breast cancer patients whose tumors overexpress erbB2 and become resistant to erbB2-targeted therapy.

## MATERIALS AND METHODS

### Reagents and antibodies

Lapatinib (L-4804) and Saracatinib (S-8906) were purchased from LC Laboratories (Woburn, MA). Akt1/2 kinase inhibitor (A6730) was purchased from Sigma Co. (St. Louis, MO). IMC-A12 (Cixutumumab), a fully human IgG1 monoclonal Ab directed against human IGF-1R, was kindly provided by ImClone Systems (New York City, NY). MISSION^®^ Non-target shRNA (SHC002), which does not target human and mouse genes, control vector (pLKO.1-ConshRNA) and pLKO.1 containing human *IGF-1R* shRNA (pLKO.1-IGF-1RshRNA, TRCN0000039675 target sequence: GCCGAAGATTTCACAGTCAAA) were purchased from Sigma. The pLKO.1 containing human *erbB3* shRNA (pLKO.1-ErbB3shRNA, TRCN0000010344 target sequence: CAATGGTAGAGTAGAGAATT) and lentivirus packaging plasmids pCMV-VSVG and pCMV-ΔA.9 were kindly provided by Dr. Haihua Gu at our Department.

Antibodies used for western blots were as follows: erbB3 (LabVision Corp., Fremont, CA); P-erbB3 (Y1289), IGF-1R, caspase-8 (1C12), caspase-3 (8G10), P-MAPK (Erk1/2) (T202/Y204), MAPK, P-Akt (S473), Akt, P-Src (Y416), Src, Survivin (6E4), and PARP rabbit mAb (Cell Signaling Technology, Inc., Beverly, MA); β-actin (Sigma Co.). All other reagents were purchased from Sigma unless otherwise specified.

### Cells and cell culture

Human breast cancer cell lines SKBR3 and BT474 were obtained from the American Type Culture Collection (Manassas, VA). The trastuzumab-resistant sublines BT474-HR20 and SKBR3-pool2, derived from BT474 and SKBR3, respectively, were described previously [41, 42]. Cell line authentication was confirmed with DNA profiling by University of Colorado Cancer Center's DNA Sequencing & Analysis Core facility in July 2010. All cell lines were free of mycoplasma contamination, which was determined by the MycoAlert™ Mycoplasma Detection Kit (Lonza Group Ltd. Basel, Switzerland) every three months. Both BT474-HR20 and SKBR3-pool2 cells were maintained in the presence of 20 μg/ml of trastuzumab. All cell lines were cultured with DMEM/F-12 (1:1) medium (Sigma) containing 10% FBS (Sigma) in a 37°C humidified atmosphere containing 95% air and 5% CO2 and split twice a week.

### Cell proliferation assay

The CellTiter96 AQ nonradioactive cell proliferation kit (Thermo Fisher Scientific Inc., Waltham, MA) was used to determine cell viability [42, 43, 53]. Briefly, cells were plated onto 96-well plates for 24 hr, and then grown in either DMEM/F12 medium as control, or the same medium containing different concentrations of lapatinib and incubated for another 72 hr. After reading all wells at 490 nm with a microplate reader, the percentages of surviving cells from each group relative to controls, defined as 100% survival, were determined by reduction of MTS.

### Specific knockdown of erbB3 or IGF-1R expression with a lentiviral system

Lentiviral production and specific knockdown of erbB3 or IGF-1R expression with a shRNA were carried out as described previously [42, 53]. In brief, the lentivirus-containing either control shRNA or erbB3/IGF-1R specific shRNA were produced in 293T cells following the standard procedure. The virus in conditioned medium were harvested, aliquot, and stored at −80°C freezer. Prior to infection, the lentivirus-containing media were thawed completely at room temperature, and mixed with a same amount of fresh medium containing polybrene (8 μg/ml). The culture media of the candidate breast cancer cells were then replaced with the lentivirus-containing media. After 24 hr, the virus-infected cells were selected with puromycin (1 μg/ml) for 48 hr, and then subjected to required experiments.

### Quantification of apoptosis

An apoptotic ELISA kit (Roche Diagnostics Corp., Indianapolis, IN) was used to quantitatively measure cytoplasmic histone-associated DNA fragments (mononucleosomes and oligonucleosomes) as previously described [42, 43, 53]. This enzyme immunoassay was performed according to the manufacturer's instructions.

### Western blot analysis

Protein expression levels were determined by western blot analysis [54–56]. Equal amounts of total cell lysates were boiled in Laemmli SDS-sample buffer, resolved by SDS-PAGE, transferred to nitrocellulose membrane (Bio-Rad Laboratories, Inc., Hercules, CA), and probed with the primary antibodies described in the figure legends. After the blots were incubated with horseradish peroxidase-labeled secondary antibody (Jackson ImmunoResearch Laboratories, Inc., West Grove, PA), the signals were detected using the enhanced chemiluminescence reagents (GE Healthcare Bio-Sciences Corp., Piscataway, NJ).

### Live/dead cell staining

Live and dead cells were visualized by Live/Dead Cell Imaging kit (Life Technologies Corp., Eugene, OR) based on a cell permeable dye for staining of live cells (green) and a cell impermeable dye for staining of the dead cells (red). Cells were seeded in a 24-well plate overnight, treated with lapatinib for 24 hr, and freshly stained with Live/Dead Imaging kit exactly following the manufacturer's instructions. Cell images were subsequently acquired under a Nikon light microscopy.

### Flow cytometric analysis of cell cycle

Flow cytometric analyses were performed to define cell cycle distribution for treated and untreated cells [54]. Briefly, cells grown in 100-mm culture dishes were harvested and fixed with 70% ethanol. Cells were then stained for total DNA content with a solution containing 50 μg/ml propidium iodide and 100 μg/ml RNase I in PBS for 30 min at 37°C. Cell cycle distribution was analyzed at the Flow Cytometry Core Facility of University of Colorado Cancer Center with a FAC Scan flow cytometer (BD Biosciences, San Jose, CA).

### Colony formation assay

Colony formation assays were performed as described previously [57]. In brief, cells at exponential growth phase were harvested with trypsin-EDTA and counted by a hemocytometer. Cells were diluted and seeded at about 1000 cells per well of a twelve-well plate. After 12 hr incubation, cells were untreated or treated with lapatinib, and then continuously cultured with the fresh media, which were changed every 3 days, for 14 days in a 37°C humidified atmosphere containing 95% air and 5% CO_2_. The cell colonies were stained for 15 min with a solution containing 0.5% crystal violet and 25% methanol, followed by three rinses with tap water to remove excess dye. The colony was defined to consisting of at least 50 cells. The colony numbers were counted by a gel documentation system (Bio-Rad Laboratories, Inc.).

### Statistical analysis

Statistical analyses of the experimental data were performed using a two-sided Student's *t* test. Significance was set at the *P* < 0.05. All values are reported at the mean +/– SD from at least three independent experiments.

## SUPPLEMENTARY FIGURES



## Abbreviations

PARP: poly (ADP-ribose) polymerase
MAPK: mitogen-activated protein kinase
PI3K: phosphoinositide 3-kinase
MBC: metastatic breast cancer
RTK: receptor tyrosine kinase
ER: estrogen receptor
PR: progestrone receptor
EGFR: epidermal growth factor receptor
HRG: heregulin
IGF-I: insulin-like growth factor-I
IGF-1R: IGF-I receptor
PTEN: phosphatase and tensin homolog
PI-3K: phosphoinositide 3-kinase
MAPK: mitogen-activated protein kinase
IHC: immunohistochemistry
ELISA: enzyme-linked immunosorbent assay
MTS: 3-(4,5-dimethylthiazol-2-yl)-5-(3-carboxymethoxyphenyl)-2-(4-sulfophenyl)-2H-tetrazolium,inner salt

## References

[1] Slamon DJ, Clark GM, Wong SG, Levin WJ, Ullrich A, McGuire WL (1987). Human breast cancer: correlation of relapse and survival with amplification of the HER-2/neu oncogene. Science.

[2] Thor AD, Schwartz LH, Koerner FC, Edgerton SM, Skates SJ, Yin S, McKenzie SJ, Panicali DL, Marks PJ, Fingert HJ, Wood WC (1989). Analysis of c-erbB-2 expression in breast carcinomas with clinical follow-up. Cancer Res.

[3] Hudis CA (2007). Trastuzumab—mechanism of action and use in clinical practice. N Engl J Med.

[4] Incorvati JA, Shah S, Mu Y, Lu J (2013). Targeted therapy for HER2 positive breast cancer. J Hematol Oncol.

[5] Mates M, Fletcher GG, Freedman OC, Eisen A, Gandhi S, Trudeau ME, Dent SF (2015). Systemic targeted therapy for her2-positive early female breast cancer: a systematic review of the evidence for the 2014 Cancer Care Ontario systemic therapy guideline. Curr Oncol.

[6] Mehta AI, Brufsky AM, Sampson JH (2013). Therapeutic approaches for HER2-positive brain metastases: circumventing the blood-brain barrier. Cancer Treat Rev.

[7] Nielsen DL, Kumler I, Palshof JA, Andersson M (2013). Efficacy of HER2-targeted therapy in metastatic breast cancer. Monoclonal antibodies and tyrosine kinase inhibitors. Breast.

[8] Patil A, Sherbet GV (2015). Therapeutic approach to the management of HER2-positive breast cancer metastatic to the brain. Cancer Lett.

[9] Nahta R, Yu D, Hung MC, Hortobagyi GN, Esteva FJ (2006). Mechanisms of Disease: understanding resistance to HER2-targeted therapy in human breast cancer. Nat Clin Pract Oncol.

[10] Chen FL, Xia W, Spector NL (2008). Acquired Resistance to Small Molecule ErbB2 Tyrosine Kinase Inhibitors. Clin Cancer Res.

[11] Rexer BN, Arteaga CL (2012). Intrinsic and acquired resistance to HER2-targeted therapies in HER2 gene-amplified breast cancer: mechanisms and clinical implications. Crit Rev Oncog.

[12] Thery JC, Spano JP, Azria D, Raymond E, Penault Llorca F (2014). Resistance to human epidermal growth factor receptor type 2-targeted therapies. European J Cancer.

[13] Amin DN, Sergina N, Lim L, Goga A, Moasser MM (2012). HER3 signalling is regulated through a multitude of redundant mechanisms in HER2-driven tumour cells. Biochem J.

[14] Garrett JT, Olivares MG, Rinehart C, Granja-Ingram ND, Sanchez V, Chakrabarty A, Dave B, Cook RS, Pao W, McKinely E, Manning HC, Chang J, Arteaga CL (2011). Transcriptional and posttranslational up-regulation of HER3 (ErbB3) compensates for inhibition of the HER2 tyrosine kinase. Proc Natl Acad Sci U S A.

[15] Wilson TR, Fridlyand J, Yan Y, Penuel E, Burton L, Chan E, Peng J, Lin E, Wang Y, Sosman J, Ribas A, Li J, Moffat J (2012). Widespread potential for growth-factor-driven resistance to anticancer kinase inhibitors. Nature.

[16] Xia W, Petricoin EF, Zhao S, Liu L, Osada T, Cheng Q, Wulfkuhle JD, Gwin WR, Yang X, Gallagher RI, Bacus S, Lyerly HK, Spector NL (2013). An heregulin-EGFR-HER3 autocrine signaling axis can mediate acquired lapatinib resistance in HER2+ breast cancer models. Breast Cancer Res.

[17] Liu L, Greger J, Shi H, Liu Y, Greshock J, Annan R, Halsey W, Sathe GM, Martin A-M, Gilmer TM (2009). Novel Mechanism of Lapatinib Resistance in HER2-Positive Breast Tumor Cells: Activation of AXL. Cancer Res.

[18] Oliveras-Ferraros C, Vazquez-Martin A, Cufi S, Torres-Garcia VZ, Sauri-Nadal T, Barco SD, Lopez-Bonet E, Brunet J, Martin-Castillo B, Menendez JA (2011). Inhibitor of Apoptosis (IAP) survivin is indispensable for survival of HER2 gene-amplified breast cancer cells with primary resistance to HER1/2-targeted therapies. Biochem Biophys Res Commun.

[19] Geyer CE, Forster J, Lindquist D, Chan S, Romieu CG, Pienkowski T, Jagiello-Gruszfeld A, Crown J, Chan A, Kaufman B, Skarlos D, Campone M, Davidson N (2006). Lapatinib plus capecitabine for HER2-positive advanced breast cancer. N Engl J Med.

[20] Cameron DA, Stein S (2008). Drug Insight: intracellular inhibitors of HER2—clinical development of lapatinib in breast cancer. Nat Clin Pract Oncol.

[21] McArthur H (2009). An overview of HER-targeted therapy with lapatinib in breast cancer. Adv Ther.

[22] Medina PJ, Goodin S (2008). Lapatinib: a dual inhibitor of human epidermal growth factor receptor tyrosine kinases. Clin Ther.

[23] Ryan Q, Ibrahim A, Cohen MH, Johnson J, Ko CW, Sridhara R, Justice R, Pazdur R (2008). FDA drug approval summary: lapatinib in combination with capecitabine for previously treated metastatic breast cancer that overexpresses HER-2. Oncologist.

[24] Yamauchi H, Cristofanilli M, Nakamura S, Hortobagyi GN, Ueno NT (2009). Molecular targets for treatment of inflammatory breast cancer. Nat Rev Clin Oncol.

[25] Berns K, Horlings HM, Hennessy BT, Madiredjo M, Hijmans EM, Beelen K, Linn SC, Gonzalez-Angulo AM, Stemke-Hale K, Hauptmann M, Beijersbergen RL, Mills GB, van de Vijver MJ (2007). A functional genetic approach identifies the PI3K pathway as a major determinant of trastuzumab resistance in breast cancer. Cancer Cell.

[26] Eichhorn PJ, Gili M, Scaltriti M, Serra V, Guzman M, Nijkamp W, Beijersbergen RL, Valero V, Seoane J, Bernards R, Baselga J (2008). Phosphatidylinositol 3-kinase hyperactivation results in lapatinib resistance that is reversed by the mTOR/phosphatidylinositol 3-kinase inhibitor NVP-BEZ235. Cancer Res.

[27] O'Brien NA, Browne BC, Chow L, Wang Y, Ginther C, Arboleda J, Duffy MJ, Crown J, O'Donovan N, Slamon DJ (2010). Activated phosphoinositide 3-kinase/AKT signaling confers resistance to trastuzumab but not lapatinib. Mol Cancer Ther.

[28] Hutchinson L (2010). Targeted therapies: Activated PI3K/AKT confers resistance to trastuzumab but not lapatinib. Nat Rev Clin Oncol.

[29] Xia W, Husain I, Liu L, Bacus S, Saini S, Spohn J, Pry K, Westlund R, Stein SH, Spector NL (2007). Lapatinib Antitumor Activity Is Not Dependent upon Phosphatase and Tensin Homologue Deleted on Chromosome 10 in ErbB2-Overexpressing Breast Cancers. Cancer Res.

[30] Wang YC, Morrison G, Gillihan R, Guo J, Ward RM, Fu X, Botero MF, Healy NA, Hilsenbeck SG, Phillips GL, Chamness GC, Rimawi MF, Osborne CK (2011). Different mechanisms for resistance to trastuzumab versus lapatinib in HER2-positive breast cancers—role of estrogen receptor and HER2 reactivation. Breast Cancer Res.

[31] Zhang S, Huang WC, Li P, Guo H, Poh SB, Brady SW, Xiong Y, Tseng LM, Li SH, Ding Z, Sahin AA, Esteva FJ, Hortobagyi GN (2011). Combating trastuzumab resistance by targeting SRC, a common node downstream of multiple resistance pathways. Nature Med.

[32] Muthuswamy SK (2011). Trastuzumab resistance: all roads lead to SRC. Nature Med.

[33] Liang K, Esteva FJ, Albarracin C, Stemke-Hale K, Lu Y, Bianchini G, Yang CY, Li Y, Li X, Chen CT, Mills GB, Hortobagyi GN, Mendelsohn J (2010). Recombinant human erythropoietin antagonizes trastuzumab treatment of breast cancer cells via Jak2-mediated Src activation and PTEN inactivation. Cancer Cell.

[34] Peiro G, Ortiz-Martinez F, Gallardo A, Perez-Balaguer A, Sanchez-Paya J, Ponce JJ, Tibau A, Lopez-Vilaro L, Escuin D, Adrover E, Barnadas A, Lerma E (2014). Src, a potential target for overcoming trastuzumab resistance in HER2-positive breast carcinoma. Br J Cancer.

[35] De Luca A, D'Alessio A, Gallo M, Maiello MR, Bode AM, Normanno N (2014). Src and CXCR4 are involved in the invasiveness of breast cancer cells with acquired resistance to lapatinib. Cell Cycle.

[36] Rexer BN, Ham AJ, Rinehart C, Hill S, Granja-Ingram Nde M, Gonzalez-Angulo AM, Mills GB, Dave B, Chang JC, Liebler DC, Arteaga CL (2011). Phosphoproteomic mass spectrometry profiling links Src family kinases to escape from HER2 tyrosine kinase inhibition. Oncogene.

[37] Huang C, Park CC, Hilsenbeck SG, Ward R, Rimawi MF, Wang YC, Shou J, Bissell MJ, Osborne CK, Schiff R (2011). beta1 integrin mediates an alternative survival pathway in breast cancer cells resistant to lapatinib. Breast Cancer Res.

[38] Jegg AM, Ward TM, Iorns E, Hoe N, Zhou J, Liu X, Singh S, Landgraf R, Pegram MD (2012). PI3K independent activation of mTORC1 as a target in lapatinib-resistant ERBB2+ breast cancer cells. Breast Cancer Res Treat.

[39] Agus DB, Akita RW, Fox WD, Lewis GD, Higgins B, Pisacane PI, Lofgren JA, Tindell C, Evans DP, Maiese K, Scher HI, Sliwkowski MX (2002). Targeting ligand-activated ErbB2 signaling inhibits breast and prostate tumor growth. Cancer Cell.

[40] Lu Y, Zi X, Zhao Y, Mascarenhas D, Pollak M (2001). Insulin-like growth factor-I receptor signaling and resistance to trastuzumab (Herceptin). [comment]. J Natl Cancer Inst.

[41] Nahta R, Yuan LX, Zhang B, Kobayashi R, Esteva FJ (2005). Insulin-like growth factor-I receptor/human epidermal growth factor receptor 2 heterodimerization contributes to trastuzumab resistance of breast cancer cells. Cancer Res.

[42] Huang X, Gao L, Wang S, McManaman JL, Thor AD, Yang X, Esteva FJ, Liu B (2010). Heterotrimerization of the growth factor receptors erbB2, erbB3, and insulin-like growth factor-i receptor in breast cancer cells resistant to herceptin. Cancer Res.

[43] Wang S, Huang X, Lee CK, Liu B (2010). Elevated expression of erbB3 confers paclitaxel resistance in erbB2-overexpressing breast cancer cells via upregulation of Survivin. Oncogene.

[44] Hurvitz SA, Hu Y, O'Brien N, Finn RS (2013). Current approaches and future directions in the treatment of HER2-positive breast cancer. Cancer Treat Rev.

[45] Jelovac D, Emens LA (2013). HER2-directed therapy for metastatic breast cancer. Oncology (Williston Park).

[46] Konecny GE, Pegram MD, Venkatesan N, Finn R, Yang G, Rahmeh M, Untch M, Rusnak DW, Spehar G, Mullin RJ, Keith BR, Gilmer TM, Berger M (2006). Activity of the dual kinase inhibitor lapatinib (GW572016) against HER-2-overexpressing and trastuzumab-treated breast cancer cells. Cancer Res.

[47] Xia W, Mullin RJ, Keith BR, Liu LH, Ma H, Rusnak DW, Owens G, Alligood KJ, Spector NL (2002). Anti-tumor activity of GW572016: a dual tyrosine kinase inhibitor blocks EGF activation of EGFR/erbB2 and downstream Erk1/2 and AKT pathways. Oncogene.

[48] Xia W, Bisi J, Strum J, Liu L, Carrick K, Graham KM, Treece AL, Hardwicke MA, Dush M, Liao Q, Westlund RE, Zhao S, Bacus S (2006). Regulation of survivin by ErbB2 signaling: therapeutic implications for ErbB2-overexpressing breast cancers. Cancer Res.

[49] Vazquez-Martin A, Oliveras-Ferraros C, Del Barco S, Martin-Castillo B, Menendez JA (2009). mTOR inhibitors and the anti-diabetic biguanide metformin: new insights into the molecular management of breast cancer resistance to the HER2 tyrosine kinase inhibitor lapatinib (Tykerb(R)). Clin Transl Oncol.

[50] Burris HA, Hurwitz HI, Dees EC, Dowlati A, Blackwell KL, O'Neil B, Marcom PK, Ellis MJ, Overmoyer B, Jones SF, Harris JL, Smith DA, Koch KM (2005). Phase I safety, pharmacokinetics, and clinical activity study of lapatinib (GW572016), a reversible dual inhibitor of epidermal growth factor receptor tyrosine kinases, in heavily pretreated patients with metastatic carcinomas. J Clin Oncol.

[51] Nahta R, Yuan LX, Du Y, Esteva FJ (2007). Lapatinib induces apoptosis in trastuzumab-resistant breast cancer cells: effects on insulin-like growth factor I signaling. Mol Cancer Ther.

[52] Chakrabarty A, Sanchez V, Kuba MG, Rinehart C, Arteaga CL (2012). Feedback upregulation of HER3 (ErbB3) expression and activity attenuates antitumor effect of PI3K inhibitors. Proc Natl Acad Sci U S A.

[53] Huang X, Gao L, Wang S, Lee CK, Ordentlich P, Liu B (2009). HDAC inhibitor SNDX-275 induces apoptosis in erbB2-overexpressing breast cancer cells via down-regulation of erbB3 expression. Cancer Res.

[54] Huang J, Wang S, Lyu H, Cai B, Yang X, Wang J, Liu B (2013). The anti-erbB3 antibody MM-121/SAR256212 in combination with trastuzumab exerts potent antitumor activity against trastuzumab-resistant breast cancer cells. Molecular Cancer.

[55] Wang S, Huang J, Lyu H, Cai B, Yang X, Li F, Tan J, Edgerton SM, Thor AD, Lee CK, Liu B (2013). Therapeutic targeting of erbB3 with MM-121/SAR256212 enhances antitumor activity of paclitaxel against erbB2-overexpressing breast cancer. Breast Cancer Res.

[56] Wang S, Huang J, Lyu H, Lee CK, Tan J, Wang J, Liu B (2013). Functional cooperation of miR-125a, miR-125b, and miR-205 in entinostat-induced downregulation of erbB2/erbB3 and apoptosis in breast cancer cells. Cell Death Dis.

[57] Liu B, Fan Z, Edgerton SM, Deng XS, Alimova IN, Lind SE, Thor AD (2009). Metformin induces unique biological and molecular responses in triple negative breast cancer cells. Cell Cycle.

