# Supplementary material for: The erbB3- and IGF-1 receptor-initiated signaling pathways exhibit distinct effects on lapatinib sensitivity against trastuzumab-resistant breast cancer cells
Source: Oncotarget. 2015 Nov 26;7(3):2921–35. doi: 10.18632/oncotarget.6404 (PMC4823081; doi:10.18632/oncotarget.6404)
Supplement: Supplementary file 1 [file oncotarget-07-2921-s001.pdf]

SUPPLEMENTARY FIGURES

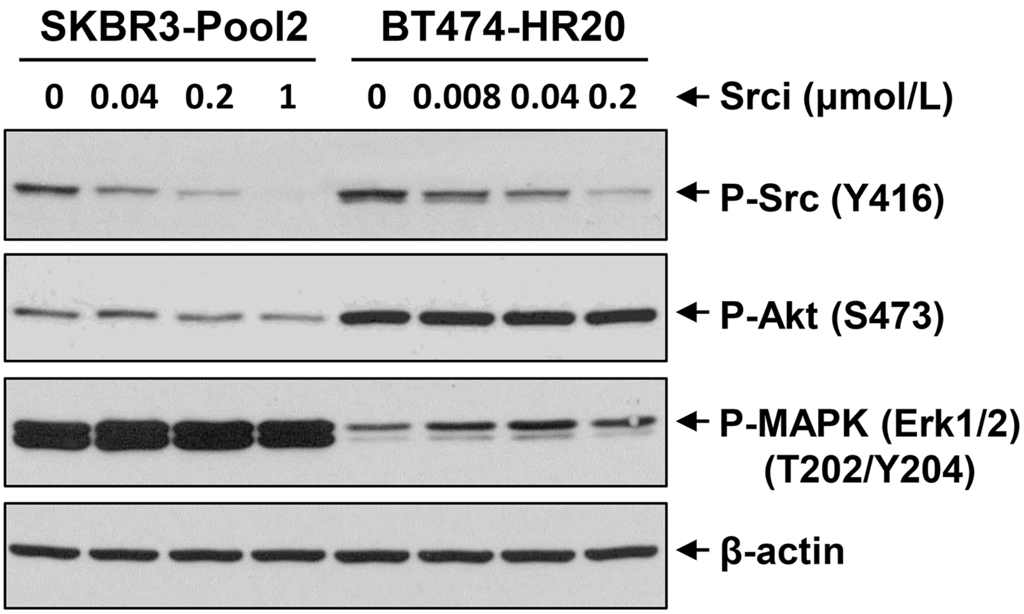

Supplementary Figure S1: The specific inhibitor of Src reduces P-Src levels of trastzumab-resistant breast cancer cells in a dose-dependent manner. SKBR3-Pool2 and BT474-HR20 cells untreated or treated with indicated concentrations of the Src inhibitor (Srci) Saracatinib for 24 hr were collected and subjected to western blot analyses of P-Src (Y416), P-Akt (S473), P-MAPK (Erk1/2) (T202/Y204), or β-actin.

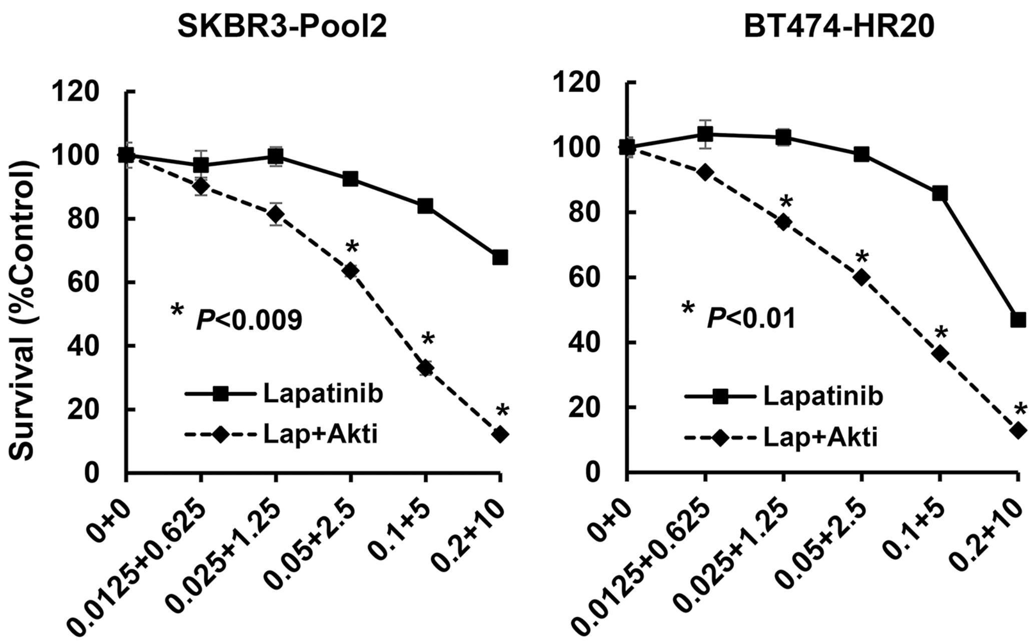

Supplementary Figure S2: The Akt inhibitor in combination with lapatinib shows a concentration dependent additional inhibitory effect on trastzumab-resistant breast cancer cells. SKBR3-Pool2 and BT474-HR20 cells were plated onto 96-well plates. After 24 hr, the culture medium was replaced with 0.1 ml fresh medium containing 0.5% FBS or the same medium containing a series of doses of lapatinib (0.0125, 0.025, 0.05, 0.1, 0.2 µmol/L) in combination with the indicated concentrations of Akti (0.625, 1.25, 2.5, 5, 10 µmol/L) for another 72 hr. The percentages of surviving cells from each cell line relative to controls, defined as 100% survival, were determined by reduction of MTS. *Bars*, SD. Data show a representative of three independent experiments.

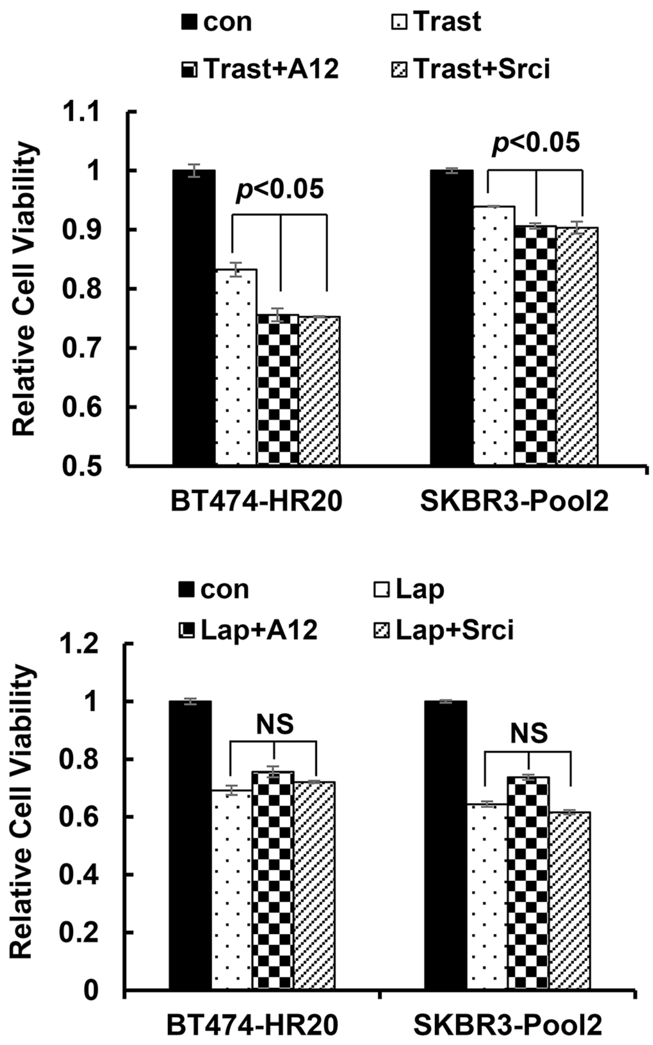

Supplementary Figure S3: Both the anti-IGF-1R Ab A12 and Src inhibitor significantly enhances trastuzumab-, but not lapatinib-mediated growth inhibition in trastzumab-resistant breast cancer cells. SKBR3-Pool2 and BT474-HR20 cells were plated onto 96-well plates. After 24 hr, the culture medium was replaced with 0.1 ml fresh medium containing 0.5% FBS or the same medium containing trastuzumab (20 µg/ml)/lapatinib (0.1 µmol/L), trastuzumab/lapatinib combined with the anti-IGF-1R Ab (Trast/Lap+A12, 10 µg/ml), trastuzumab/lapatinib combined with Src inhibitor (Trast /Lap+Srci, 0.2 µmol/L) for another 72 hr. The viability of cells from each cell line relative to controls, defined as 1, were determined by reduction of MTS. *Bars*, SD. Data show a representative of three independent experiments.
